# Dengue score: a proposed diagnostic predictor for pleural effusion and/or ascites in adults with dengue infection

**DOI:** 10.1186/s12879-016-1671-3

**Published:** 2016-07-08

**Authors:** Suhendro Suwarto, Leonard Nainggolan, Robert Sinto, Bonita Effendi, Eppy Ibrahim, Maulana Suryamin, R. Tedjo Sasmono

**Affiliations:** Division of Tropical and Infectious Diseases, Department of Internal Medicine, Faculty of Medicine, Universitas Indonesia, Cipto Mangunkusumo National Hospital, Jakarta, Indonesia; Department of Epidemiology, Faculty of Public Health, Universitas Indonesia, Jakarta, Indonesia; Department of Internal Medicine, Persahabatan Hospital, Jakarta, Indonesia; Eijkman Institute of Molecular Biology, Jl. Diponegoro 69, Jakarta, 10430 Indonesia

**Keywords:** Albumin, Ascites, AST ratio, Dengue score, Hematocrit, Platelet, Pleural effusion

## Abstract

**Background:**

There are several limitations in diagnosing plasma leakage using the World Health Organization (WHO) guidelines of dengue hemorrhagic fever. We conducted a study to develop a dengue scoring system to predict pleural effusion and/or ascites using routine laboratory parameters.

**Methods:**

A prospective observational study was carried out at Cipto Mangunkusumo Hospital and Persahabatan Hospital, Jakarta, Indonesia. Dengue-infected adults admitted on the third febrile day from March, 2010 through August, 2015 were included in the study. A multivariate analysis was conducted to determine the independent diagnostic predictors of pleural effusion and/or ascites and to convert the prediction model into a scoring system.

**Results:**

A total of 172 dengue-infected adults were enrolled in the study. Of the 172 patients, 101 (58.7 %) developed pleural effusion and/or ascites. A multivariate analysis was conducted to determine the independent diagnostic predictors of pleural effusion and/or ascites in dengue-infected adults. The predictors were scored based on the following calculations: hemoconcentration ≥15.1 % had a score of 1 (OR, 3.11; 95 % CI, 1.41–6.88), lowest albumin concentration at critical phase ≤3.49 mg/dL had a score of 1 (OR, 4.48; 95 % CI, 1.87–10.77), lowest platelet count ≤49,500/μL had a score of 1 (OR, 3.62; 95 % CI, 1.55–8.49), and elevated ratio of AST ≥2.51 had a score of 1 (OR 2.67; 95 % CI, 1.19–5.97). At a cut off of ≥ 2, the Dengue Score predicted pleural effusion and/or ascites diagnosis with positive predictive value of 79.21 % and negative predictive value of 74.63 %. This prediction model is suitable for calibration and good discrimination.

**Conclusions:**

We have developed a Dengue Score that could be used to identify pleural effusion and/or ascites and might be useful to stratify dengue-infected patients at risk for developing severe dengue.

## Background

Two-fifths of the world’s populations in tropical and subtropical countries are at risk of dengue. An estimated 500,000 people with dengue infection require hospitalization each year [1, 2]. Based on a 20-year registry of dengue infection age based-epidemiology in Indonesia, there appears to be an epidemiological shift in the age of affected individuals. Since 1999, a steady decline in incidence was observed for children aged 5 to 14 years (the age group with highest DHF incidence, historically). The decline surpassed the increasing incidence of affected individuals aged over 15 years [3].

The presence of plasma leakage, one of the characteristics of dengue hemorrhagic fever (DHF), is the prominent cause of severe dengue [4, 5]. The timely diagnosis and management of plasma leakage are very important [4]. Fujimoto et al. reported that 5.7 % of patients with plasma leakage developed cardiorespiratory dysfunction, and the mortality rate reached 7.3 % [6]. Chairulfatah et al. reported 6 % severe bleeding in 1300 pediatric and adult DHF cases in Indonesia [7].

The World Health Organization (WHO) guidelines defined plasma leakage with the occurrence of hemoconcentration and/or hypoalbuminemia and/or serous effusion [2, 5, 8–10]. However, there are several limitations in diagnosing plasma leakage using these criteria. In clinical practice, clinicians often detect pleural effusion and/or ascites in patients with elevated hematocrit values of less than 20 %, a cut off used to define hemoconcentration as recommended by WHO (1, 8, 9). Moreover, previous studies showed albumin level <3.5 g/dL in dengue fever (DF) and DHF patients, which is in contrast with WHO criteria that use 3.5 g/dL to differentiate patients with and without plasma leakage [2, 11–14]. Compared with hematocrit and albumin level, pleural effusions and/or ascites as visualized by ultrasonography (USG) are highly sensitive and specific for determining plasma leakage [15–18]. However, USG is not widely available in resource limited areas [19]. Despite the currently accepted laboratory parameters for determining plasma leakage, pathophysiologically there are associations between plasma leakage identified by the presence of pleural effusions and/or ascites with thrombocytopenia and elevated hepatic transaminase levels [20, 21]. To the best of our knowledge, there are no studies reporting the cut off point of the lowest platelet count and elevated ratio of transaminase levels at the critical phase for diagnosing pleural effusion and/or ascites. Similarly, no reports regarding the cut off point of hyponatremia, which is another common manifestation of plasma leakage [10], is available.

Here, we conducted a study to develop a dengue scoring system to predict pleural effusion and/or ascites using laboratory parameters, such as the degree of hemoconcentration, lowest albumin concentration at the critical phase, degree of hypoalbuminemia, lowest platelet count, elevated ratio of aspartate aminotransferase (AST), alanine aminotransferase (ALT), and sodium concentration at critical phase. This Dengue Score can be used to identify pleural effusions and/or ascites, which are better indicators of plasma leakage, to stratify dengue-infected patients at risk of developing severe dengue.

## Methods

### Patients and study design

This was a prospective observational study conducted at the ward of Cipto Mangunkusumo and Persahabatan Hospitals, Jakarta, Indonesia. Dengue-infected patients admitted on the third febrile day from March 2010 through August 2015 were included in the study. Adult patients aged 14 years or above were included. Dengue infection was diagnosed when acute febrile patients with axillary temperature above 37.5 °C were proven positive for dengue nonstructural protein (NS) 1 antigen test (Standard Diagnostics, Korea). The exclusion criteria included pregnant women, patients with comorbidities and patients who were not willing to participate.

### Dengue virus (DENV) serotyping and clinical laboratory examination

DENV serotypes were determined using both conventional RT-PCR according to method by Lanciotti et al. [22] and Simplexa Dengue real-time RT-PCR (Focus Diagnostics, Cypress, CA, USA) [23]. Virus RNA was extracted directly from 140 μl serum samples using QIAamp viral RNA mini kit (Qiagen, Hilden, Germany), performed according to the manufacturer’s instructions. The resulting RNAs were subjected to RT-PCR assays. The patients’ characteristics, onset of fever, and clinical findings were recorded for each subject upon arrival at the emergency department for enrollment. A complete blood count, including hematocrit level, platelet count, and albumin level were performed daily until the subjects met the discharge criteria based on the WHO guidelines [5]. Hepatic transaminases (AST and ALT) levels and sodium concentrations were measured twice during the critical phase as defined by 24–48 h after defervescence. Abdominal USG to detect pleural effusions and/or ascites using conventional ultrasound devices with a 3.5 MHz transducer were performed by a certified radiologist 24 h after defervescence [5]. To validate the USG results, another certified radiologist examined the ultrasonogram of every subject.

**Fig. 1:**
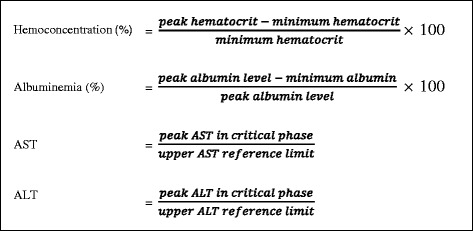
Formulae for calculating the degrees of hemoconcentration, albuminemia, and the ratios of AST and ALT

The degree of hemoconcentration (%) was calculated according to the formula of subtracting the peak hematocrit with the minimum hematocrit recorded, then dividing that value by the minimum hematocrit recorded and multiplying by 100 (Fig. 1). The degree of hypoalbuminemia (%) was calculated according to the formula of subtracting the peak albumin with the minimum albumin level recorded, divided by the peak albumin level and multiplying by 100 (Fig. 1). Elevated ratios of AST or ALT were calculated by dividing the peak AST or ALT in the critical phase by the upper reference limit (Fig. 1).

### Statistical analysis

The sample size of the study was based on an estimated 48 % prevalence of hemoconcentration in subjects with pleural effusion and/or ascites [24]. Assuming an odds ratio of 1.75, with α = 0.05 and β = 0.20, the required total sample size was calculated to be 168 patients.

A bivariate analysis with student’s *t*-test for normally distributed data and a Mann Whitney test for non-parametric data were performed to identify variables that had a significant association with the presence of pleural effusion and/or ascites. To determine the appropriate cut off values of each significant variable (*p* < 0.05), the receiving operating characteristic (ROC) curve analysis was used to obtain the best sensitivity and specificity for a diagnosis of pleural effusion and/or ascites. These best cut off values were used to classify the subjects into categorical variables. All of the categorical variables had a significant effect based on bivariate analysis and were then submitted to multivariate analysis.

We entered the variables into a multiple regression analysis using a backward selection algorithm to estimate the coefficients and independent diagnostic predictors of pleural effusion and/or ascites. The coefficients were converted into a simplified risk score system using published methods [25–27]. The performance of the model was assessed by the Hosmer Lemeshow test for its calibration and by an area under receiving operating characteristic curve (AROC) for its discriminating ability. Statistical analyses were performed using the STATA statistical software version 12 (Stata Corp., College Station, TX, USA).

## Results

### Patients’ characteristics and clinical laboratory parameters

A total of 172 dengue infected-patients were enrolled in the study; the patients had a median age of 22 (interquartile range = 11) years old, and 89 (51.7 %) were men. Most patients (34.3 %) were infected with DENV-2 . Of the 172 patients, 101 (58.7 %) developed a pleural effusion and/or ascites. The characteristics of the subjects are presented in Table 1.

**Table 1:** Dengue infected-patients: comparison of patients with and without pleural effusion and/or ascites

Variable	Without pleural effusion and/or ascites (*n* = 71)	With pleural effusion and/or ascites (*n* = 101)
Sex (*n*, male/female)	32/39	57/44
Age (year)^b^	21 (11)	22 (12)
Dengue serotype		
DENV-1 (%)	20 (28.17)	25 (24.75)
DENV-2 (%)	27 (38.03)	32 (31.69)
DENV-3 (%)	17 (23.94)	30 (29.70)
DENV-4 (%)	7 (9.86)	14 (13.86)
Degree of hemoconcentration (%)^b^	12.67 (7.03)*	18.92 (10.81)*
Lowest albumin concentration at critical phase (g/dL)^b^	3.70 (0.35)*	3.31 (0.46)*
Degree of hypoalbuminemia (%)^b^	11.36 (9.00)*	20.63 (12.67)*
Lowest platelet count (x 1,000/μL)^b^	75.00 (66.00)*	32.00 (31.50)*
Elevated ratio of AST^b^	1.80 (1.46)*	3.11 (2.84)*
Elevated ratio of ALT^b^	0.88 (1.00) *	1.22 (1.16)*
Sodium concentration at critical phase (mEq/L)^a^	135.37 ± 5.83	134.05 ± 5.09

*ALT* alanine aminotransferase, *AST* aspartate aminotransferase

^a^data presented as the mean ± standard deviation

^b^data presented as median (interquartile range)

*p < 0.05 by Mann Whitney test

Six of seven diagnostic predictors of pleural effusion and/or ascites were found to have a significant association with the presence of pleural effusion and/or ascites (Table 1). We determined the best cut off value of those six significant variables using receiving operating characteristic (ROC) curve analysis (Table 2) as the reference for classifying those predictors into categorical variables. There are two albumin related diagnostic predictors of pleural effusion and/or ascites, i.e., the lowest albumin concentration at the critical phase (≤3.49) and the degree of hypoalbuminemia (>15.6 %; Table 2). Those two variables have a similar AROC. For practicality, we selected the lowest albumin concentration at the critical phase as the predictor of pleural effusion and/or ascites because it is only measured once.

**Table 2:** Receiver operator curve characteristics for significant parameters in predicting pleural effusion and/or ascites diagnosis and cut off value for maximum sensitivity and specificity

Variable	AROC (95 % CI)	Cut off	Sens (%)	Spe (%)	LR+	LR-
Degree of hemoconcentration (%)	0.74 (0.67–0.82)	≥15.10	67.3	69	2.17	0.47
Lowest albumin concentration at critical phase (mg/dL)	0.78 (0.72–0.86)	≤3.49	70	81.7	3.78	0.37
Degree of hypoalbuminemia (%)	0.76 (0.69–0.84)	≥15.6	74.2	72.3	2.68	0.36
Lowest platelet count (/μL)	0.83 (0.76–0.89)	≤49,500	72.3	76.1	3.02	0.36
Elevated ratio of AST	0.77 (0.70–0.85)	≥2.51	70.1	74.29	2.72	0.40
Elevated ratio of ALT	0.63 (0.54–0.71)	≥1.01	64.9	57.7	1.51	0.60

*ALT* alanine aminotransferase, *AST* aspartate aminotransferase, *AROC* area under receiving operating characteristic curve, *LR*- negative likelihood ratio, *LR*+ positive likelihood ratio, *CI* confident interval, *Sens* sensitivity, *Spe* specificity

The bivariate analyses to predict pleural effusion and/or ascites using the five variables are presented in Table 3. All variables with a significant effect based on the bivariate analysis were then subjected to a multivariate analysis. The following variables were found to be independent diagnostic predictors of pleural effusion and/or ascites: degree of hemoconcentration ≥15.1 %, lowest albumin concentration at the critical phase ≤3.49 g/dL, lowest platelet count ≤49,500/μL, and elevated ratio of AST ≥2.51 (Table 3).

**Table 3:** Final multiple logistic regression model to predict pleural effusion and/or ascites

Variable	Bivariate analysis		Multivariate adjusted			
	Odds ratio (95 % CI)	*p* Value	Coefficient	Odds ratio (95 % CI)	*p* Value	Dengue Score
Degree of hemoconcentration ≥ 15.1 %	4.30 (2.25–8.22)	<0.001	1.13	3.11 (1.41–6.88)	0.005	1
Lowest albumin concentration at critical phase ≤3.49 g/dL	10.07 (4.83–21.01)	<0.001	1.50	4.48 (1.87–10.77)	0.001	1
Lowest platelet count ≤ 49,500/μL	8.28 (4.12–16.64)	<0.001	1.28	3.62 (1.55–8.49)	0.003	1
Elevated ratio of AST ≥ 2.51	4.94 (2.54–9.62)	<0.001	0.98	2.67 (1.19–5.97)	0.017	1
Elevated ratio of ALT ≥ 1.01	2.50 (1.33–4.70)	0.004				

*ALT* alanine aminotransferase, *AST* aspartate aminotransferase, *CI* confident interval

### Scoring system development

The Dengue Score was generated by dividing each multivariate logistic regression coefficient by the smallest coefficient in the model (elevated ratio of AST ≥2.51). The score weights were calculated by dividing the coefficient/standard error (2.38) as follows: degree of hemoconcentration >15.1 %, lowest albumin concentration at critical phase ≤3.49 mg/dL, lowest platelet count ≤49,500/μL, resulting in quotients of 1.17, 1.40, and 1.24, respectively. The quotients were rounded to the nearest integer, resulting in score weights of one for each variable (Table 3).

### Performance of the score

**Fig. 2:**
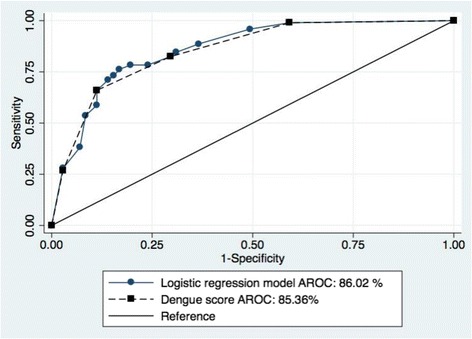
Comparison of area receiver operating characteristic (AROC) curves between the logistic regression model and Dengue Score

The Dengue Score was tested at different cut off values, and the results are shown in Table 4. At a cut off of ≥ 2, the score had a sensitivity of 82.47 %, specificity of 70.42 %, positive predictive value (PPV) 79.21 %, negative predictive value (NPV) of 74.63 %, and correctly predicted pleural effusion and/or ascites diagnosis at a rate of 77.38 %. The AROC of the logistic regression probability model was 86.02 % (95 % CI: 80.3–91.8 %), and the Dengue Score AROC was 85.36 % (95 % CI: 79.5–91.2 %). The significance levels of the AROC of the 2 models were comparable (*p* = 0.34; Fig. 2). The model fitted the observed data according to the Hosmer-Lemeshow goodness-of-fit test (*p* =0.362).

**Table 4:** The sensitivity and specificity of the Dengue Score at different cut offs

Cut off	Sensitivity	Specificity	PPV	NPV	Correctly Classified
≥1	98.97 %	40.85 %	69.57 %	96.67 %	74.40 %
≥2	82.47 %	70.42 %	79.21 %	74.63 %	77.38 %
≥3	65.98 %	88.73 %	88.89 %	65.63 %	75.60 %
≥4	26.80 %	97.10 %	92.86 %	49.29 %	56.55 %

*NPV* negative predictive value, *PPV* positive predictive value

## Discussion

This is the first Indonesian study to determine a scoring system to predict pleural effusion and/or ascites in adult patients with dengue infection. Michels et al. and Balasubramanian et al. showed that pleural effusion and/or ascites detection by USG is superior to clinical and laboratory parameters for diagnosing plasma leakage [15, 16]. In this study, we tried to determine independent diagnostic predictors of pleural effusion and/or ascites and to convert the prediction model into a scoring system that could be practically used because USG is not routinely available in all healthcare facilities. Hematocrit, albumin, hepatic transaminases, platelet count, and sodium concentrations are laboratory parameters that are routinely checked in the management of dengue patients as recommended by the WHO [2, 5, 10]. Therefore, the scoring system to predict the plasma leakage that was represented in this study by the detection of pleural effusion and/or ascites would be highly applicable in various healthcare settings where USG is not available.

As reported in other study, there is a significant difference in the degree of hemoconcentration in dengue infected patients with and without pleural effusions and/or ascites [28]. We found a similar result, with a degree of hemoconcentration of 12.67 % (inter-quartile range - IQR 7.03 %) in patients without pleural effusion/ascites and 18.92 % (IQR 10.81 %) in patients with pleural effusion/ascites. Translating this finding, DHF would be underdiagnosed by physicians relying only on hematocrit as a diagnostic criterion. The ROC analysis showed that compared with classically used cut off point of degree of hemoconcentration ≥20 % as suggested by the WHO [5, 10], a cut off point ≥15.1 % gives a better performance for predicting the presence of pleural effusion and/or ascites. Our study suggests the use of the lowest cut off point of hemoconcentration to define plasma leakage to minimize the risk of under-diagnosing patients at risk of severe dengue. A degree of hemoconcentration ≥15.1 % was given a score of 1 in the final Dengue Score.

The WHO defines a significantly decreased albumin >0.5 g/dL from baseline or <3.5 g/dL as indirect evidence of plasma leakage [2, 10]. However, two publications reported low albumin levels (<3.5 g/dL) in patients with DF and DHF [11, 12]. Jagadishkumar et al. [11] and Itha et al. [12] found median albumin levels of 3.1 g/dL to 3.37 g/dL in DF and 2.7 g/dL to 3.23 g/dL in DHF groups based on the WHO criteria. Roy et al. [13] reported that mean albumin levels in dengue-infected patients without and with warning signs were 3.48 and 3.34 g/dL, respectively. We observed that the use of two albumin-related parameters, i.e., the lowest albumin concentration at the critical phase and the degree of hypoalbuminemia, for predicting a diagnosis of pleural effusion and/or ascites may not be conclusive. We found that ≤3.49 g/dL and ≥15.6 % were the best cut off points for the two parameters, respectively. We suggest using degree of hypoalbuminemia as a percentage to detect plasma leakage, similar to the concept of degree of hemoconcentration. However, due to the similarity in AROC between the 2 parameters, we selected the lowest albumin concentration at critical phase, which is more practical in daily practice, as albumin is only measured once at critical phase. In DHF, hypoalbuminemia results from the loss of albumin due to the occurrence of plasma leakage. This phenomenon is similar with the pathophysiology of pleural effusion and/or ascites in DHF, thereby explaining the association between the two conditions [29]. Similar with degree of hemoconcentration, lowest albumin concentration at critical phase ≤3.49 g/dL was given a score of 1 in the final Dengue Score.

Thrombocytopenia has been accepted as a major sign of plasma leakage in dengue-infected patients. Damage to platelets, which possibly contribute to thrombocytopenia in dengue patients, results in the release of vascular endothelial growth factor (VEGF) which in turn is responsible for the occurrence of pleural effusions and/or ascites [30, 31]. As shown in Table 1, we found a significant difference in the medians between patient with and without pleural effusion and/or ascites. Using cut off point of ≤49,500/μL, it gave the best AROC among all the variables tested. This variable was given a score of 1 in the final Dengue Score.

Our data show that liver injury, manifested by an elevated ratio of hepatic transaminases, was frequently present in adult patients with dengue infection. Liver cells are one of the targets of dengue viruses. The liver dysfunction was mild to moderate, presenting primarily as elevations of hepatic transaminases, with significantly higher AST elevation in patients with pleural effusion and/or ascites [32, 33]. Consistent with the findings of Mahmuduzzaman et al. [34], we found a greater increase of AST compared with ALT in dengue patients. In the multivariate analysis, an elevated ratio of ALT failed to show a significant association with pleural effusion and/or plasma leakage. The exact significance of the greater elevation of AST compared with ALT in dengue might be because of the excess release of AST from damaged myocytes during dengue infections [35]. Further research is needed to confirm this hypothesis. An elevated ratio of AST is a laboratory marker of dengue severity that has been proposed by several researchers but has not gained much attention, as reflected by its absence in the plasma leakage criteria of the WHO [2, 5, 10]. By calculating the transaminase elevation ratio, we are attempting to address the problem of the difference between laboratories in the reference limits of the AST [36]; therefore, this diagnostic predictor variable could be universally used across laboratories.

This study has limitation that should be considered. This study was conducted in patients without comorbidities; therefore, the generalization should be re-tested in population with comorbidities, especially in those with comorbidities that potentially influencing the levels/concentration of predictors’ variables, e.g., chronic kidney disease and chronic liver disease. Future study is needed to validate the Dengue Score and to investigate the effect of the application of this Dengue Score in various healthcare facilities managing dengue-infected patients.

## Conclusions

We have developed a Dengue Score to predict the diagnosis of a pleural effusion and/or ascites in adults with dengue infection. This score might provide early identification of patients who are at risk for developing severe dengue.

## Abbreviations

ALT, alanine aminotransferase; AROC, area under receiving operating characteristic curve; AST, aspartate aminotransferase; DF, dengue fever; DHF, dengue hemorrhagic fever; LR-, negative likelihood ratio; LR+, positive likelihood ratio; NPV, negative predictive value; NS, nonstructural protein; OR, odds ratio; PPV, positive predictive value; ROC, receiving operating characteristic; Sens, sensitivity; Spe, specificity; USG, ultrasonography; VEGF, vascular endothelial growth factor; WHO, World Health Organization
